# Supplementary material for: Active site specificity profiling datasets of matrix metalloproteinases (MMPs) 1, 2, 3, 7, 8, 9, 12, 13 and 14
Source: Data Brief. 2016 Feb 22;7:299–310. doi: 10.1016/j.dib.2016.02.036 (PMC4777984; doi:10.1016/j.dib.2016.02.036)
Supplement: Supplementary file 10 — Supplementary material [file mmc10.zip › WebPICS_hMMP2_T_1%/PICS_results.html]

 

PICS results


- # About
- # Manual
- # Analysis
- # Results

- Redraw
- Seqlogo
- Dependency
- Coop

**PICS analysis of protease: hMMP2\_T\_1%**  
642 cleavage sites analyzed (total was 672)  
PICS library made with (T)rypsin, (G)luC or (C)hymotrypsin: T  

|  |  |
| --- | --- |
| Positional occurences    **Values > 10 % are shown** (table for total and relative (in %) values) | Occurences relative to natural abundance   **Values > 2 fold natural abundance are shown** (table) |
|  |  |

  
  
Results (including this html file) in one compressed file for local use.
  
  
Total occurences (open csv)

|  |  |  |  |  |  |  |  |  |  |  |  |  |
| --- | --- | --- | --- | --- | --- | --- | --- | --- | --- | --- | --- | --- |
|  | P6 | P5 | P4 | P3 | P2 | P1 | P1prime | P2prime | P3prime | P4prime | P5prime | P6prime |
| A | 44 | 54 | 64 | 62 | 169 | 107 | 25 | 59 | 116 | 90 | 71 | 60 |
| C | 7 | 5 | 10 | 18 | 19 | 7 | 12 | 8 | 3 | 2 | 6 | 6 |
| D | 23 | 21 | 28 | 2 | 24 | 36 | 1 | 3 | 22 | 35 | 55 | 58 |
| E | 36 | 33 | 29 | 9 | 39 | 32 | 15 | 17 | 29 | 47 | 31 | 56 |
| F | 8 | 14 | 22 | 18 | 9 | 15 | 23 | 30 | 17 | 29 | 23 | 16 |
| G | 24 | 51 | 45 | 18 | 78 | 104 | 3 | 21 | 132 | 65 | 40 | 61 |
| H | 15 | 13 | 15 | 11 | 5 | 23 | 2 | 31 | 18 | 20 | 23 | 25 |
| I | 14 | 29 | 33 | 71 | 13 | 2 | 109 | 69 | 18 | 36 | 40 | 24 |
| K | 1 | 7 | 17 | 0 | 4 | 0 | 1 | 12 | 3 | 14 | 8 | 14 |
| L | 30 | 39 | 43 | 67 | 34 | 15 | 302 | 97 | 20 | 55 | 59 | 56 |
| M | 14 | 12 | 14 | 8 | 6 | 8 | 24 | 22 | 8 | 12 | 9 | 8 |
| N | 19 | 17 | 17 | 13 | 15 | 74 | 3 | 20 | 21 | 18 | 24 | 22 |
| P | 35 | 39 | 50 | 171 | 8 | 56 | 1 | 2 | 10 | 46 | 53 | 61 |
| Q | 23 | 29 | 31 | 8 | 40 | 42 | 24 | 34 | 22 | 30 | 32 | 33 |
| R | 1 | 0 | 13 | 0 | 2 | 0 | 2 | 4 | 4 | 7 | 7 | 2 |
| S | 34 | 47 | 42 | 20 | 103 | 60 | 16 | 44 | 103 | 42 | 42 | 37 |
| T | 20 | 28 | 31 | 15 | 17 | 24 | 8 | 53 | 36 | 32 | 36 | 38 |
| V | 26 | 30 | 46 | 84 | 20 | 8 | 51 | 88 | 44 | 50 | 67 | 54 |
| W | 8 | 4 | 1 | 4 | 1 | 8 | 9 | 6 | 5 | 4 | 5 | 1 |
| Y | 13 | 14 | 18 | 3 | 6 | 8 | 11 | 22 | 11 | 8 | 11 | 10 |

  
  
Relative occurences in % (open csv)

|  |  |  |  |  |  |  |  |  |  |  |  |  |
| --- | --- | --- | --- | --- | --- | --- | --- | --- | --- | --- | --- | --- |
|  | P6 | P5 | P4 | P3 | P2 | P1 | P1prime | P2prime | P3prime | P4prime | P5prime | P6prime |
| A | 6.9 | 8.4 | 10.0 | 9.7 | 26.3 | 16.7 | 3.9 | 9.2 | 18.1 | 14.0 | 11.1 | 9.3 |
| C | 1.1 | 0.8 | 1.6 | 2.8 | 3.0 | 1.1 | 1.9 | 1.2 | 0.5 | 0.3 | 0.9 | 0.9 |
| D | 3.6 | 3.3 | 4.4 | 0.3 | 3.7 | 5.6 | 0.2 | 0.5 | 3.4 | 5.5 | 8.6 | 9.0 |
| E | 5.6 | 5.1 | 4.5 | 1.4 | 6.1 | 5.0 | 2.3 | 2.6 | 4.5 | 7.3 | 4.8 | 8.7 |
| F | 1.2 | 2.2 | 3.4 | 2.8 | 1.4 | 2.3 | 3.6 | 4.7 | 2.6 | 4.5 | 3.6 | 2.5 |
| G | 3.7 | 7.9 | 7.0 | 2.8 | 12.1 | 16.2 | 0.5 | 3.3 | 20.6 | 10.1 | 6.2 | 9.5 |
| H | 2.3 | 2.0 | 2.3 | 1.7 | 0.8 | 3.6 | 0.3 | 4.8 | 2.8 | 3.1 | 3.6 | 3.9 |
| I | 2.2 | 4.5 | 5.1 | 11.1 | 2.0 | 0.3 | 17.0 | 10.7 | 2.8 | 5.6 | 6.2 | 3.7 |
| K | 0.2 | 1.1 | 2.6 | 0.0 | 0.6 | 0.0 | 0.2 | 1.9 | 0.5 | 2.2 | 1.2 | 2.2 |
| L | 4.7 | 6.1 | 6.7 | 10.4 | 5.3 | 2.3 | 47.0 | 15.1 | 3.1 | 8.6 | 9.2 | 8.7 |
| M | 2.2 | 1.9 | 2.2 | 1.2 | 0.9 | 1.2 | 3.7 | 3.4 | 1.2 | 1.9 | 1.4 | 1.2 |
| N | 3.0 | 2.6 | 2.6 | 2.0 | 2.3 | 11.5 | 0.5 | 3.1 | 3.3 | 2.8 | 3.7 | 3.4 |
| P | 5.5 | 6.1 | 7.8 | 26.6 | 1.2 | 8.7 | 0.2 | 0.3 | 1.6 | 7.2 | 8.3 | 9.5 |
| Q | 3.6 | 4.5 | 4.8 | 1.2 | 6.2 | 6.5 | 3.7 | 5.3 | 3.4 | 4.7 | 5.0 | 5.1 |
| R | 0.2 | 0.0 | 2.0 | 0.0 | 0.3 | 0.0 | 0.3 | 0.6 | 0.6 | 1.1 | 1.1 | 0.3 |
| S | 5.3 | 7.3 | 6.5 | 3.1 | 16.0 | 9.3 | 2.5 | 6.9 | 16.0 | 6.5 | 6.5 | 5.8 |
| T | 3.1 | 4.4 | 4.8 | 2.3 | 2.6 | 3.7 | 1.2 | 8.3 | 5.6 | 5.0 | 5.6 | 5.9 |
| V | 4.0 | 4.7 | 7.2 | 13.1 | 3.1 | 1.2 | 7.9 | 13.7 | 6.9 | 7.8 | 10.4 | 8.4 |
| W | 1.2 | 0.6 | 0.2 | 0.6 | 0.2 | 1.2 | 1.4 | 0.9 | 0.8 | 0.6 | 0.8 | 0.2 |
| Y | 2.0 | 2.2 | 2.8 | 0.5 | 0.9 | 1.2 | 1.7 | 3.4 | 1.7 | 1.2 | 1.7 | 1.6 |

  
  
Occurences relative to natural abundance   

|  |  |  |  |  |  |  |  |  |  |  |  |  |
| --- | --- | --- | --- | --- | --- | --- | --- | --- | --- | --- | --- | --- |
|  | P6 | P5 | P4 | P3 | P2 | P1 | P1prime | P2prime | P3prime | P4prime | P5prime | P6prime |
| A | 0.8 | 1.0 | 1.2 | 1.2 | 3.2 | 2.0 | 0.5 | 1.1 | 2.2 | 1.7 | 1.3 | 1.1 |
| C | 0.8 | 0.6 | 1.2 | 2.1 | 2.2 | 0.8 | 1.4 | 0.9 | 0.4 | 0.2 | 0.7 | 0.7 |
| D | 0.7 | 0.6 | 0.8 | 0.1 | 0.7 | 1.0 | 0.0 | 0.1 | 0.6 | 1.0 | 1.6 | 1.7 |
| E | 0.8 | 0.8 | 0.7 | 0.2 | 0.9 | 0.7 | 0.3 | 0.4 | 0.7 | 1.1 | 0.7 | 1.3 |
| F | 0.3 | 0.6 | 0.9 | 0.7 | 0.4 | 0.6 | 0.9 | 1.2 | 0.7 | 1.2 | 0.9 | 0.6 |
| G | 0.5 | 1.1 | 1.0 | 0.4 | 1.7 | 2.3 | 0.1 | 0.5 | 2.9 | 1.4 | 0.9 | 1.3 |
| H | 1.0 | 0.9 | 1.0 | 0.7 | 0.4 | 1.6 | 0.1 | 2.1 | 1.2 | 1.4 | 1.6 | 1.7 |
| I | 0.4 | 0.8 | 0.9 | 1.9 | 0.3 | 0.1 | 2.8 | 1.8 | 0.5 | 0.9 | 1.0 | 0.6 |
| K | 0.0 | 0.2 | 0.4 | 0.0 | 0.1 | 0.0 | 0.0 | 0.3 | 0.1 | 0.4 | 0.2 | 0.4 |
| L | 0.5 | 0.6 | 0.7 | 1.1 | 0.5 | 0.2 | 4.9 | 1.6 | 0.3 | 0.9 | 1.0 | 0.9 |
| M | 0.9 | 0.8 | 0.9 | 0.5 | 0.4 | 0.5 | 1.5 | 1.4 | 0.5 | 0.8 | 0.6 | 0.5 |
| N | 0.7 | 0.6 | 0.6 | 0.5 | 0.6 | 2.8 | 0.1 | 0.8 | 0.8 | 0.7 | 0.9 | 0.8 |
| P | 1.2 | 1.3 | 1.7 | 5.7 | 0.3 | 1.9 | 0.0 | 0.1 | 0.3 | 1.5 | 1.8 | 2.0 |
| Q | 0.9 | 1.1 | 1.2 | 0.3 | 1.6 | 1.6 | 0.9 | 1.3 | 0.9 | 1.2 | 1.3 | 1.3 |
| R | 0.0 | 0.0 | 0.4 | 0.0 | 0.1 | 0.0 | 0.1 | 0.1 | 0.1 | 0.2 | 0.2 | 0.1 |
| S | 0.8 | 1.1 | 1.0 | 0.5 | 2.5 | 1.4 | 0.4 | 1.1 | 2.5 | 1.0 | 1.0 | 0.9 |
| T | 0.6 | 0.8 | 0.9 | 0.4 | 0.5 | 0.7 | 0.2 | 1.6 | 1.1 | 0.9 | 1.1 | 1.1 |
| V | 0.6 | 0.7 | 1.0 | 1.9 | 0.5 | 0.2 | 1.1 | 2.0 | 1.0 | 1.1 | 1.5 | 1.2 |
| W | 1.1 | 0.6 | 0.2 | 0.6 | 0.2 | 1.1 | 1.3 | 0.8 | 0.7 | 0.6 | 0.7 | 0.2 |
| Y | 0.7 | 0.8 | 1.0 | 0.2 | 0.3 | 0.4 | 0.6 | 1.2 | 0.6 | 0.4 | 0.6 | 0.5 |
